# The effect of a medication reconciliation program in two intensive care units in the Netherlands: a prospective intervention study with a before and after design

**DOI:** 10.1186/s13613-018-0361-2

**Published:** 2018-02-07

**Authors:** Liesbeth B. E. Bosma, Nicole G. M. Hunfeld, Rogier A. M. Quax, Edmé Meuwese, Piet H. G. J. Melief, Jasper van Bommel, SiokSwan Tan, Maaike J. van Kranenburg, Patricia M. L. A. van den Bemt

**Affiliations:** 1 Department of Pharmacy, Haga Teaching Hospital, Els Borst-Eilersplein 275, 2545 CH The Hague, The Netherlands; 2 Apotheek Haagse Ziekenhuizen, PO Box 43100, 2504 AC The Hague, The Netherlands; 3 Department of Hospital Pharmacy, Erasmus University Medical Center, PO Box 2040, 3000 CA Rotterdam, The Netherlands; 4 Department of Intensive Care, Erasmus University Medical Center, PO Box 2040, 3000 CA Rotterdam, The Netherlands; 5 Department of Internal Medicine, Maasstad Teaching Hospital, Maasstadweg 21, 3079 DZ Rotterdam, The Netherlands; 6 Department of Intensive Care, Haga Teaching Hospital, PO Box 43100, 2504 AC The Hague, The Netherlands; 7 Department of Public Health, Erasmus University Medical Center, PO Box 2040, 3000 CA Rotterdam, The Netherlands; 8 Department of Hospital Pharmacy, Gelre Hospitals, PO Box 9014, 7300 DS Apeldoorn, The Netherlands

**Keywords:** Medication reconciliation, Intensive care unit, Pharmacist, Adverse drug event, Cost–benefit analysis

## Abstract

**Background:**

Medication errors occur frequently in the intensive care unit (ICU) and during care transitions. Chronic medication is often temporarily stopped at the ICU. Unfortunately, when the patient improves, the restart of this medication is easily forgotten. Moreover, temporal ICU medication is often unintentionally continued after ICU discharge. Medication reconciliation could be useful to prevent such errors. Therefore, the aim of this study was to determine the effect of medication reconciliation at the ICU.

**Methods:**

This prospective 8-month study with a pre- and post-design was carried out in two ICU settings in the Netherlands. Patients were included when they used ≥ 1 chronic medicine and when the ICU stay exceeded 24 h. The intervention consisted of medication reconciliation by pharmacists at the moment of ICU admission and prior to ICU discharge. Medication transfer errors (MTEs) were collected and the severity of potential harm of these MTEs was measured, based on a potential adverse drug event score (pADE = 0; 0.01; 0.1; 0.4; 0.6). Primary outcome measures were the proportions of patients with ≥ 1 MTE at ICU admission and after discharge. Secondary outcome measures were the proportions of patients with a pADE score ≥ 0.01 due to these MTEs, the severity of the pADEs and the associated costs. Odds ratio and 95% confidence intervals were calculated, by using a multivariate logistic regression analysis.

**Results:**

In the pre-intervention phase, 266 patients were included and 212 in the post-intervention phase. The proportion of patients with ≥ 1 MTE at ICU admission was reduced from 45.1 to 14.6% (OR_adj_ 0.18 [95% CI 0.11–0.30]) and after discharge from 73.9 to 41.2% (OR_adj_ 0.24 [95% CI 0.15–0.37]). The proportion of patients with a pADE ≥ 0.01 at ICU admission was reduced from 34.8 to 8.0% (OR_adj_ 0.13 [95% CI 0.07–0.24]) and after discharge from 69.5 to 36.2% (OR_adj_ 0.26 [95% CI 0.17–0.40]). The pADE reduction resulted in a potential net cost–benefit of € 103 per patient.

**Conclusions:**

Medication reconciliation by pharmacists at ICU transfers is an effective safety intervention, leading to a significant decrease in the number of MTE and a cost-effective reduction in potential harm.

*Trial registration* Dutch trial register: NTR4159, 5 September 2013, retrospectively registered

## Background

Intensive care unit (ICU) patients are at risk for medication errors and adverse drug events (ADEs) because of the complexity of their conditions, the need for urgent interventions and the considerable workload fluctuation of the ICU staff [1, 2]. In addition, certain hospital processes carry a high risk for medication errors. One of these processes is the transition of care. Approximately 60% of the medication errors occur at care transitions [3]. Lee et al. [4] showed that clinically significant medication transfer errors (MTEs) occur in 6 out of 10 patients when being shifted from one hospital ward to another. The main cause of MTEs is incorrect or incomplete communication, although healthcare providers spend much time trying to validate the accuracy of patient medication at these interfaces of care [5].

The critical illness at the time of admission usually causes long-term medication used at home to be temporarily withheld in the ICU patient [5]. Unfortunately, when the patient improves, the restart of this medication is easily forgotten. In addition, medication initiated during the ICU stay for short-term use, such as gastric acid secretion inhibitors [6–8] and antipsychotics [9–13], is often inadvertently continued after ICU and even after hospital discharge [14].

Among critically ill patients, the medication error rate ranges from 1.2 to 947 errors per ICU patient days and is an important cause of patient morbidity and mortality. About 10% of these medication errors are thought to result in an ADE [15]. Various interventions have been studied to reduce medication errors on the ICU. In a systematic review by Manias et al., medication reconciliation at ICU admission was one of the four interventions demonstrating a reduction in medication errors [16]. A small number of studies suggest that the incidence of medication errors during and after hospitalization can be reduced by medication reconciliation at ICU discharge [17–19]. However, these studies have limitations such as small sample size, failure to differentiate between intentional and unintentional discrepancies and lack of assessment of potential clinical impact and/or severity of discrepancies.

Studies combining medication reconciliation at ICU admission and at ICU discharge are lacking. Therefore, we designed a pre- and post-intervention study on the effect of medication reconciliation by a pharmacist on the proportion of patients with medication transfer errors (MTEs) at admission to and at discharge from the ICU. Furthermore, the effect on the number, severity and cost of adverse drug events, as were estimated based on the MTE (i.e., potential ADE), was studied.

## Methods

### Aim

The aim of this study was to determine the effect of a medication reconciliation program performed by pharmacists on the proportion of patients with MTEs both at ICU admission and ICU discharge. In addition, the severity of potential harm of these MTEs was measured, based on a potential adverse drug event score (pADE = 0; 0.01; 0.1; 0.4; 0.6). Furthermore, a cost–benefit analysis was performed.

### Study design

The TIM (*T*ransfer *I*CU and *M*edication reconciliation) study was a prospective 8-month intervention study with a before and after design in two Dutch hospitals. The pre-intervention phase consisted of 14 weeks of usual care [General Teaching Hospital (GTH): January–April 2013 and University Hospital (UH): February–May 2014]. After a 2-week implementation period, the intervention program with medication reconciliation by a pharmacist at both ICU admission and ICU discharge started. The post-intervention phase consisted of 14 weeks (GTH: May–September 2013, UH: July–October 2014). A detailed description of the study protocol is published elsewhere [19].

### Setting and study population

The study was carried out in the Haga Teaching hospital in The Hague (GTH; 18 ICU beds) and the Erasmus University Medical Center in Rotterdam (Erasmus MC; UH; 32 ICU beds).

Patients were included when they used at least one medicine at home and when the ICU length of stay exceeded 24 h. An ICU discharge and readmission within 24 h was counted as the same ICU admission.

At discharge, patients were included if they were included in the admission part of the study and if they survived until at least 24 h after ICU discharge.

Exclusion criteria were: transfer to another hospital, both admission and discharge within the same weekend (Friday 17:00 until Monday 8:30) and patient’s inability to be counseled in Dutch or English. None of the patients of the pre-intervention group were part of the post-intervention group.

**Fig. 1:**
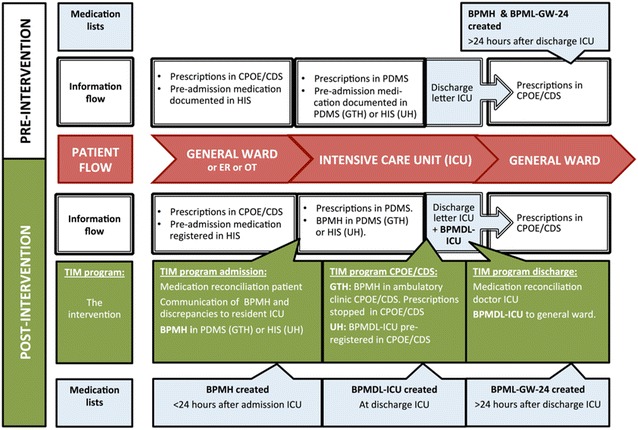
Study procedure pre- and post-intervention. *BPMDL-ICU* best possible ICU medication discharge list, *BPMH* best possible medication history, *BPML-GW24* best possible general ward medication list 24 h after ICU discharge, *CPOE/CDS system* computerized physician order entry systems with clinical decision support, *ER* emergency room, *HIS* hospital information system, *ICU* intensive care unit, *OT* operating theater, *PDMS* patient data monitoring system, *TIM* Transfer ICU and Medication reconciliation program

Since this study did not affect patients’ integrity, a waiver from the Zuid Holland Medical Ethics committee (METC) and the Erasmus MC METC was obtained. This waiver is in line with Dutch trial legislation. Data collection complied with privacy regulations. Figure 1 gives an overview of the study procedures.

### Pre-intervention phase: usual care

Upon ICU admission, the ICU physician collected information about pre-admission medication and registered this in the patient data management system of the ICU (PDMS). The GTH ICU used Metavision (Itémedical BV, Tiel, The Netherlands) and the UH ICU used Care Suite 8.2 (PICIS Inc., Wakefield, MA, USA). The ICU discharge letter contained information about medication in use at discharge. Sometimes pre-admission medication and/or suggestions for medication use after discharge were registered.

After transfer, the physician of the admitting ward had to transcribe medication orders from the discharge letter to the hospital electronic patient records.

### The intervention

After ICU admission, a *b*est *p*ossible *m*edication *h*istory (BPMH) was constructed, based on a medication history of 6 months from the community pharmacy, available hospital medication information and a medication verification interview with the patient and/or a representative. On the medication history of the community pharmacy, the latest date of filling was documented, as well as the date of the medication was due to be finished. Based on this list, we interviewed the patient and/or caretaker asking for all medication currently in use, the used dose, etc. By combining pharmacy record information with the patient information, we were able to get the best possible medication list. This is common practice in medication reconciliation, based on the WHO High 5 s program [21]. The BPMH included drug name, dosage, frequency and route—as well as an analysis of discrepancies between the medication used at home and prescribed at ICU admission [22]. The BPMH was documented in the PDMS and presented to the ICU physician responsible for the patient, helping him or her by explaining the effect of the medicine. We supported the physician to make the right decision on stopping or continuing. The ICU pharmacists also used the BPMH during their patient rounds at the ICU.

Shortly before ICU discharge, the ICU pharmacist made a discharge medication summary based on the BPMH and medication used prior to ICU discharge. For each medicine, the ICU physician was prompted for possible recommendations (i.e., restart, stop and continue). During reconciliation of this list with the doctor, the pharmacist helped the doctor to make the right advice for the ward. As a result, a best possible ICU medication discharge list (BPMDL-ICU) was made. This was sent as an annex of the ICU discharge letter to the physician of the receiving ward.

The medication was pre-registered by the pharmacist in the Computerized Physician Order Entry/Clinical Decision Support (CPOE/CDS) system of the general ward the patient was sent to [20]. By doing so, the ward doctor was supported by the pharmacist in the transcribing process. To prescribe the proper after ICU medication, at the right frequency, the right dose and route, the ward doctor only had to check the already pre-registered medication and, if found appropriate, simply authorize this pre-registered medication.

### Outcome measures

The primary outcomes were the proportions of patients with ≥ 1 MTE 24 h after ICU admission and 24 h after ICU discharge.

An MTE at admission was defined as an unintentional discrepancy between BPMH and medication prescribed 24 h after admission to the ICU. An MTE at discharge was defined as an unintentional discrepancy between the actual medication chart of the patient and the best possible general ward medication list best possible general ward medication list 24 h after the ICU discharge (BPML-GW24). This BPML-GW24 was based on the BPMH, on information in the electronic patient records of the hospital and the PDMS, on medication prescribed in the CPOE/CDS and, whenever necessary, on interviewing the physician on the ward afterward.

Data collection was performed by trained ICU pharmacists. Whether a discrepancy was intentional or not was based on information documented in the HIS or the PDMS, information given during the medication reconciliation, the ICU standards of care and the ICU pharmacist’s interpretation of the situation. Whenever necessary, the physician on the ward was interviewed afterward. In this way, we gave the doctor the opportunity to correct the error made. Two pharmacists performed a crosscheck on the data. Subsequently, all identified MTE underwent a validity check during the pADE assessment of the MTEs (see below).

The secondary outcomes were the proportions of patients with a pADE score ≥ 0.01 due to an MTE at ICU admission and at ICU discharge. A pADE was defined as an MTE that could potentially cause harm and/or clinical deterioration and was based on the methodology described by Nesbit et al. [23, 24] using the following categories for pADE scores: 0 (zero likelihood of an ADE expected by the MTE), 0.01 (very low likelihood of an ADE), 0.1 (low likelihood of an ADE), 0.4 (medium likelihood of an ADE) or 0.6 (high likelihood of an ADE).

All MTEs at ICU admission and discharge were presented blinded and in randomized order to two assessors: one hospital pharmacist/clinical pharmacologist and one internist/clinical pharmacologist in training, both with ICU experience, who independently from each other, gave a pADE score for each MTE, based on clinical data of the patient. For MTEs that were given a different pADE severity score in the assessments, the assessors reached consensus in a meeting.

We measured a total pADE score for every patient by summing up the individual pADE scores. These pADE scores reflected potential harm per patient in the following way: pADE = 0 (no harm expected), 0.01 ≤ pADE > 0.1 (very low likelihood of an ADE), 0.1 ≤ pADE > 0.4 (low likelihood of an ADE), 0.4 ≤ pADE > 0.6 (medium likelihood of an ADE) and pADE ≥ 0.6 (high likelihood of an ADE).

### Cost–benefit analysis

The cost avoidance of the TIM program was determined by subtracting the average pADE score per patient post-intervention from the average pADE score pre-intervention. This difference was multiplied by the number of patients post-intervention and the relative cost of an ADE.

The relative ADE cost price was set at € 1079. This was derived from a study by Rottenkolber and was indexed to 2014 [25].

Costs incurred by the reconciliation process were restricted to labor costs of the pharmacist. The direct time spent on this intervention was calculated using the bottom-up approach, i.e., measuring the number of minutes spent per patient by the pharmacist in a representable group of patients. These minutes were multiplied with the cost price of one minute of labor and a marginal markup percentage to account for indirect labor time (43%) [26]. The cost price of one minute was valued €1.18, based on standardized costs per minute [27].

The costs per patient were multiplied with the total number of included patients and the percentages of availability of the BPMH and the BPMDL-ICU, respectively. All costs were based on 2014 Euro cost data.

#### Sensitivity analysis

A one-way sensitivity analysis was performed for known variables in order to determine the effect of varying these estimates on the cost–benefit analysis.

The time spent on the intervention was varied by ± 50%. Salary costs were varied by using the highest senior hospital pharmacist scale, the lowest point on a basic pharmacist scale and the salary costs of a pharmacy technician with 7 years of experience. For ADE costs, we used the study by Bates et al. [28] as alternative to the study by Rottenkolber [25], thus varying the costs to €7177 per ADE. Finally, the ADE probability was varied by ± 50% [23, 24].

### Data collection

Data were collected from the hospital electronic patient records, PDMS records, CPOE/CDS medication charts, BPMH, BPMDL-ICU and BPML-GW24. All data were collected in MS Access 2007 (version 2007, Microsoft Nederland BV, Amsterdam).

We collected the following TIM intervention characteristics: availability and quality of the BPMH and the BPMDL-ICU and the used sources (i.e., patient list, electronic patient file, medication brought from home and pharmacy medication history). The quality of the BPMH and the BPMDL-ICU was set at A, B or C [20]. Quality A was defined as a reliable reconciliation (based on a recent, reliable community pharmacy medication list and a reliable verification with patient and/or his representative), quality B as an intermediate and quality C as a sub-optimal reconciliation.

The following medication information was collected: name of medicine, dose form, medication group [30], dose and frequency; prescribed in the PDMS within 24 h after admission; prescribed in the CPOE/CDS within 24 h after the ICU discharge. All discrepancies had an intended or non-intended score, a pADE score and a discrepancy type (omission, medication added, different dose or substitution).

### Data analysis

#### Sample size

The primary outcome of this study was the proportion of patients with ≥ 1 MTE at admission and discharge from the ICU. Based on the literature, the expected proportion of patients with MTE between wards within one hospital is 62% [4]. Based on a conservative interpretation of this study, we took a proportion of 30% in our study. With an estimated 50% reduction in errors due to the intervention, an alpha of 0.05 and a power of 0.80 calculated the sample size was 133. With an estimated mortality of 35%, in each measurement phase 205 patients should be included. We estimated extra loss of 30% due to the ICU stay less than 24 h and another 35% loss due to weekend ICU stay. Based on the number of ICU admissions per year, this resulted in a study period per intervention arm of 7 weeks for Erasmus MC and 8 weeks for Haga. To be on the safe side and to measure during a robust intervention period, we doubled the number of weeks. Therefore, a pre- and post-intervention period of 14 weeks was chosen. Based on an alpha of 0.05 and a power of 0.80, the calculated sample size was 205 patients per measurement phase for the primary outcome of this study. Based on the number of admissions per year and the potential loss due to ICU stays of less than 24 h and admission and discharge in one weekend, a pre- and post-intervention period of 14 weeks was chosen [20].

#### Statistical analysis

All data were analyzed with SPSS Statistics (version 24, IBM Corp., New York).

Patient characteristics pre- and post-intervention were compared using the two sample *t* test for continuous normally distributed variables, Mann–Whitney *U* test for continuous non-normally distributed variables and Chi-square test for categorical variables.

For the primary (the proportions of patients with ≥ 1 MTE at ICU admission and at ICU discharge) and secondary outcomes (the proportions of patients with a pADE score of ≥ 0.01 at ICU admission and ICU discharge), adjusted odds ratios and 95% confidence intervals (95% CI) were calculated by using a multivariate logistic regression analysis. Potential confounders were selected based on a univariate analysis (*p* < 0.20) and were retained in the multivariate model when they changed the beta-coefficient with more than 10%.

## Results

### Patient, intervention and MTE characteristics

We included 264 patients in the pre-intervention and 212 in the post-intervention phase at admission and 203 and 177 at discharge. The two populations differed with respect to APACHE IV score [29], percentage of surgical patients and specialty (Table 1).

**Table 1:** Patient characteristics

Characteristic	Pre-intervention phase (*n* = 264)	Post-intervention phase (*n* = 212)	*p* value
Age (years), mean (SD)	61.3 (14.7)	61.8 (13.4)	0.70^a^
ICU, GTH	106 (40.2%)	83 (39.2%)	0.88^b^
Sex, female (%)	98 (37.1%)	89 (42.0%)	0.28^b^
Days on ICU, median (range)	3 (1–67)	3.5 (1–75)	0.56^c^
Acute admission, *n* (%)	168 (63.6%)	125 (59.0%)	0.30^b^
Surgical, *n* (%)	94 (35.6%)	105 (49.5%)	0.02^b^
APACHE IV, mean (SD)	79.1 (32.3)	73.22 (32.9)	0.056^a^
Died in ICU^d^, *n* (%)	61 (23.1%)	35 (16.5%)	0.10^b^
Specialty, *n* (%)			0.01^b^
Internal medicine	26 (9.8%)	23 (10,8%)	
Cardiology	58 (22.0%)	30 (14.2%)	
Neurosurgery	14 (5.3%)	21 (9.9%)	
Pulmonology	16 (6.1%)	16 (7.5%)	
Neurology	31 (11.7%)	16 (7.5%)	
Surgery	75 (28.4%)	66 (31.1%)	
Gastroenterology	23 (8.7%)	14 (6.6%)	
Hematology	13 (4.9%)	6 (2.8%)	
Rest	8 (3.0%)	20 (9.4%)	
Admitted from, *n* (%)			0.45^b^
Emergency room	68 (25.8%)	46 (21.7%)	
Community	1 (0.4%)	4 (1.9%)	
Ward	97 (36.7%)	79 (37.3%)	
Operating theater	88 (33.6%)	76 (35.8%)	
Other hospital	10 (3.8%)	7 (3.3%)	
Admission at night^e^, *n* (%)	86 (32.6%)	70 (33.0%)	0.67^b^
Admission in weekend, *n* (%)	68 (25.8%)	44 (20.8%)	0.22^b^
Discharge at night^e,f^, *n* (%)	13 (6.4%)	12 (6.8%)	0.88^b^
Discharge in weekend^f^, *n* (%)	35 (17.2%)	28 (15.8%)	0.71^b^
No of medications on			
BPMH (median)	5 (1–24)	6 (1–20)	0.69^c^
BPMDL-ICU (median)	–	11 (1–25)	
BPML-GW24 (median)	11 (1–25)	10.0 (4–23)	0.61^c^
Total no of medications on			
BPMH	1655	1359	
BPML-GW24	2212	1886	

*BPMDL-ICU* best possible ICU medication discharge list, *BPMH* best possible medication history, *BPML-GW24* best possible general ward medication list 24 h after ICU discharge

^a^*T* test

^b^Chi-square test

^c^Mann–Whitney *U* test

^d^1 person pre-intervention died within 24 h after ICU discharge

^e^Night = 18.00–06.00 h

^f^Percentage based on ICU survivors, *n* = 202

Table 2 shows the intervention characteristics. In 87.3% of the cases, it was possible to generate a BPMH. Of the patients discharged from the ICU 158 (89.3%) had a BPMH and 122 (68.9%) had a BPMDL-ICU. We found 174 (98.3%) of the patients having at least one medication reconciliation performed at the ICU.

**Table 2:** Intervention characteristics

Admission	Patients (*n* = 212)
BPMH available (*n*, %)	185 (87.3%)
Quality BPMH	
A = optimal	129 (60.8%)
B = no (proper) conversation	79 (37.3%)
C = poor quality	4 (1.9%)
Reconciliation BPMH with	
Patient	76 (35.8%)
Caregiver	60 (28.3%)
Minutes per BPMH (incl. + 43%^a^)	24.0 (34.3)
Used sources	
List from patient	9 (4.2%)
Emergency room electronic patient file	18 (8.4%)
Home medication	11 (5.2%)
Community pharmacy	190 (89.6%)
Other institution	24 (11.3%)

Discharge	Patients (*n* = 177)
BPMDL-ICU available (*n*, %)	122 (68.9%)
BPMH available^b^ (*n*, %)	158 (89.3%)
BPMH and/or BPMDL-ICU available (*n*, %)	174 (98.3%)
Quality BPMDL-ICU	
A = optimal	119 (67.2%)
B = no (proper) conversation	4 (2.3%)
C = poor quality	1 (0.6%)
Minutes per BPMDL-ICU (incl. + 43%^a^)	29.4 (42.0)

*BPMDL-ICU* best possible ICU medication discharge list, *BPMH* best possible medication history, *BPML-GW24* best possible general ward medication list 24 h after ICU discharge

^a^Adjusted for indirect labor time

^b^The percentage patients who survived the ICU and were discharged to the general ward and had a BPMH available

Table 3 shows the MTE types. Omission was the most frequently occurring reason for an MTE in all groups.

**Table 3:** MTE characteristics

MTE types	Pre-intervention phase	Post-intervention phase	*p* value
	MTE = 206	MTE = 39	
*Admission*			
Omission	163 (79.1%)	25 (64.1%)	0.11
Drug added	10 (4.9%)	1 (2.6%)	
Different dose	28 (13.6%)	10 (25.6%)	
Substitution	4 (1.9%)	2 (5.1%)	
No discrepancy	1 (0.5%)	1 (2.6%)	

	MTE = 399 MTE	MTE = 122	
*Discharge*			
Omission	288 (72.2%)	88 (72.1%)	0.83
Drug added	39 (9.8%)	10 (8.2%)	
Different dose	51 (12,8%)	15 (12.3%)	
(Re)start	1 (0.3%)	0 (0.0%)	
Substitution	20 (5.0%)	9 (7.4%)	

*MTE* medication transfer error

### Primary outcome: patients with MTE

At admission 45.1% of the patients had at least 1 MTE pre-intervention compared to 14.6% in the post-intervention phase, a reduction of 67.6% (OR_adj_ 0.18 (95% CI 0.11–0.30), adjusted for APACHE IV).

At discharge 73.9% of the patients had at least 1 MTE pre-intervention, compared to 41.2% in the post-intervention phase, a reduction of 44.2% (OR_adj_ 0.24 [95% CI 0.15–0.37], adjusted for APACHE IV) (Table 4).

**Table 4:** Medication transfer errors (MTE) and potential adverse drug event (pADE) outcomes

MTE and pADE outcomes	Pre-intervention phase	Post-intervention phase	OR_adj_^a^ [CI 95%]
	Patients = 264	Patients = 212	
ICU admission			
Patients with ≥ 1 MTE (*n*, %)	119 (45.1%)	31 (14.6%)	0.18 [0.11–0.30]
Patients with ≥ 0.01 pADE (*n*, %)	92 (34.8%)	17 (8.0%)	0.13 [0.07–0.24]
Without harm (pADE = 0)	27 (22.7%)	14 (45.2%)	
Very low harm expected (0.01 ≤ pADE > 0.1)	35 (29.4%)	6 (19.4%)	
Low harm expected (0.1 ≤ pADE > 0.4)	45 (37.8%)	7 (22.6%)	
Medium harm expected (0.4 ≤ pADE > 0.6)	7 (5.9%)	3 (9.7%)	
High harm expected (pADE ≥ 0.6)	5 (4.1%)	1 (3.2%)	
MTE (*n*, per patient)	206 (0.78)	39 (0.18)	
pADE (*n*, per patient)	12.58 (0.05)	2.77 (0.01)	
Medications with MTE (% of all medications)	12.3%	2.9%	
Medications with ≥ 0.01 pADE (*n*, % of all medications)	146 (8.7%)	20 (1.5%)	
Total prevented MTE^b^ (*n*, per patient)		126.4 (0.60)	
Total prevented pADE^c^ (*n*, per patient)		7.33 (0.03)	

	Patients = 203	Patients = 177	
ICU discharge			
Patients with ≥ 1 MTE (*n*, %)	150 (73.9%)	73 (41.2%)	0.24 [0.15–0.37]
Patients with ≥ 0.01 pADE (*n*, %)	141 (69.5%)	64 (36.2%)	0.26 [0.17–0.40]
Without harm (pADE = 0)	9 (6.0%)	9 (12.3%)	
Very low harm expected (0.01 ≤ pADE > 0.1)	33 (22.0%)	28 (38.4%)	
Low harm expected (0.1 ≤ pADE > 0.4)	56 (37.3%)	25 (34.3%)	
Medium harm expected (0.4 ≤ pADE > 0.6)	33 (21.9%)	6 (8.2%)	
High harm expected (pADE ≥ 0.6)	19 (12.7%)	5 (7%)	
MTE (*n*, per patient)	399 (1.97)	122 (0.69)	
pADE (*n*, per patient)	41.97 (0.21)	9.55 (0.05)	
Medications with MTE (% of all medications)	17.9%	6.4%	
Medications with ≥ 0.01 pADE (*n*, % of all medications)	14.9% (333)	5.4% (102)	
Total prevented MTE^b^ (*n*, per patient)		225.9 (1.28)	
Total prevented pADE^c^ (*n*, per patient)		26.59 (0.15)	

*MTE* medication transfer error, *pADE* potential adverse drug event

^a^Adjusted for APACHE IV

^b^Average MTE per patient at intervention subtracted by score pre-intervention and multiplied with number of patients at intervention

^c^Average pADE score per patient at the intervention subtracted by score pre-intervention and multiplied with number of patients at intervention

### Secondary outcome: patients with potential harm

The proportion of patients with a pADE ≥ 0.01 at ICU admission was reduced from 34.8 to 8.0%, a reduction of 77.0% (OR 0.21 [95% CI 0.14–0.33] and OR_adj_ 0.13 [95% CI 0.07–0.24] adjusted for APACHE IV). Five patients (1.9%) had a high (≥ 0.6) pADE pre-intervention compared to 1 (0.5%) in the post-intervention phase.

At discharge, the proportion of patients with a pADE ≥ 0.01 was reduced from 69.5% to 36.2%, a reduction of 47.9% (OR 0.26 [95% CI 0.17–0.40] and OR_adj_ 0.26 [95% CI 0.17–0.40] adjusted for APACHE IV). Nineteen patients (9.4%) had a high (≥ 0.6) pADE pre-intervention compared to 5 (2.8%) in the post-intervention phase (Table 4).

“Appendix” provides examples of MTE with different pADE scores.

### Cost–benefit analysis

Table 5 shows a positive cost–benefit ratio of 2.48, leading to a potential net cost–benefit of €103 per patient. Costs of the intervention were € 7476 at admission and € 7256 at discharge. At admission 7.33 pADEs were prevented, leading to a cost avoidance of € 7911 at admission. At discharge 26.59 pADEs were prevented, leading to a cost avoidance of € 28,687. In the sensitivity analysis, the cost–benefit remained positive in all scenarios. The largest variance was found in costs assigned to an ADE (ADE range, adjusted to 2014: € 1079 (Rottenkolber [25])–€7177 (Bates [25]).

**Table 5:** Cost–benefit and sensitivity analysis

Cost–benefit analysis		
Calculation	Costs and benefits	Outcome^a^
1.	Costs of SERVICE (Pharmacist labor)	
	Admission	−€ 7476
	Discharge	−€ 7256
2.	Cost avoidance	
	Admission	€ 7911
	Discharge	€ 28,687
3. (= 2–1)	Net cost–benefit	
	During intervention period	€ 21,868
	Per patient (at admission)	€ 103
4. (= 2:1)	Cost–benefit ratio	2.48

Sensitivity analysis		
Variable	Variation	Cost–benefit ratio
Time	+ 50% minutes per intervention	1.66
	− 50% minutes per intervention	4.96
Salary	Highest point on hospital pharmacist scale	2.22
	Lowest point on hospital pharmacist scale	3.55
	Pharmacy technician (7th year)	7.73
ADE probability	− 50%	1.24
	50%	3.73
ADE cost	Based on Bates et al. [28]	16.52

*ADE* adverse drug event

^a^Based on 2014 Euro cost data

## Discussion

In this prospective intervention study, the proportion of patients with ≥ 1 medication transfer error (MTE) at ICU admission was reduced from 45.1 to 14.6% and at discharge from 73.9 to 41.2%. At admission 7.33 potential adverse drug events (pADEs) were prevented and at discharge 26.59 pADEs. The cost–benefit ratio of 2.48 indicates that €1000 (= 14.2 h) spent on a pharmacist for medication reconciliation at the ICU will avoid a cost of €2480. This equals a potential net cost saving of €103 per patient, suggesting that medication reconciliation by a pharmacist is cost beneficial.

Although the MTE and pADE burden was the highest at discharge of the ICU, the TIM intervention was most effective at ICU admission. There are two possible explanations for this. First, at ICU admission, the BPMH was given to the ICU physician who could directly act by changing the prescribed medication in the PDMS, while at ICU discharge the intervention was more indirect since the BPMDL-ICU was not reviewed with the physician of the admitting ward, but with the ICU physician. Second, the ICU discharge service was delivered less frequently (87.3 versus 70.1%), as for this part of the TIM program, it was more challenging to reach the ICU physician in time; the actual time frame at discharge was in general short, whereas the discharge reconciliation process was complex and therefore time-consuming.

Compared to Provonost et al. [17] who found a reduction in almost 94% MTE at ICU discharge, the effect of our TIM program seems far less with 41.2%. However, Provonost’s study measured the effect at the information written on the ICU discharge order and their medication reconciliation was not based on information from the home pharmacy.

Strengths of our study include the prospective study design and the carefully designed TIM intervention program. Other strengths of our study were the use of unintentional discrepancies (MTE) and their potential for harm (pADE) as outcome measures as well as a cost–benefit analysis. Finally, our study was performed at two different settings, which makes our results robust.

This study has a number of limitations as well. First, the study did not include a hospital setting in which the PDMS and the CPOE/CDS systems were an integrated part of the same electronic patient record. Therefore, a certain part of our MTEs could be due to transcription problems. However, because of all medication changes in the ICU, the ward physician has to review all medication after ICU discharge anyway, regardless of the CPOE/CDS situation. Second, clear documentation of reasons why home medication was withheld was generally lacking. This made the discrepancy assessment complicated. We overcame this problem by a thorough methodology of strict scoring and crosschecking. Third, the study measured potential ADEs, rather than actual ADEs and the extent of long-term effects of the harm caused by the MTE could not be determined, since we didn’t follow-up after hospital discharge. Fourth, as our cost–benefit analysis was based on reduction in potential ADE, instead of actual ADE, our cost–benefit analysis was preliminary. For this reason, we used the most conservative ADE price and we performed a sensitivity analysis, which remained positive in all scenarios. Finally, a before-after design is less robust than a randomized controlled design, but for this safety intervention a randomized controlled design is not feasible.

In our opinion, the success of our program was based on a combination of three elements: (1) focus on the transition, (2) a structured approach for the collection of medication history and discrepancy analysis, combined with (3) the ICU pharmacist knowledge and skills. We assume that this program can be as successful in other ICUs. Although we do not know the single impact of the different elements of our program, we think for the medication reconciliation part at ICU admission this could probably be performed by trained pharmacy technicians as well. However, as we found the discharge part far more complicated to properly perform (e.g., being able to interpret all ICU protocols, continuation of high risk medication, restarting a patient’s medication), we find that a pharmacist with specific ICU knowledge and understanding of the discharge process is necessary for the discharge process. As we found our intervention to have a positive cost–benefit ratio, we recommend hospitals to consider having an ICU pharmacist for medication reconciliation at the ICU.

Our results indicate that more focus on post-ICU care is necessary to further reduce inappropriate medication discontinuation and unintentional continuation of ICU medication after critical illness. This is in line with Bell et al. [5], who stated that discharge from the ICU is a time when longer-term treatment goals should be contemplated and usual medications should be restarted or reconsidered.

Future research should focus on further development of the combined ICU medication reconciliation process, for example by introducing ICT tools. Furthermore, the clinical and financial effect of medication reconciliation should be measured based on actual harm instead of MTEs and pADEs.

## Conclusions

Medication reconciliation by a pharmacist at ICU admission and discharge was an effective safety intervention, improving the continuity of care for the ICU patient, leading to a significant decrease in the number of MTEs and a cost-effective reduction in potential harm.

## Appendix

See Table 6.

**Table 6:** Detailed information on several identified medication transfer errors (MTE) and their potential for harm (Nesbit score [1]), both at ICU admission and/or ICU discharge

Patient information	Medication	Type of error	Description of transfer error	Possible harm (nesbit score [1])
*High harm expected (nesbit = 0.6)*				
Male, 80 years old, APACHE IV score = 74, 2 days on ICU	*Vancomycin* ICU dosage: 750 mg IV once daily [based on TDM (patient on dialysis)]	ICU discharge: omission	Vancomycin was not continued in a patient with pericarditis (*Staphylococcus epidermis*). The ICU advice (continue vancomycin for 6 weeks) was missed	ICU discharge: 0.6
Female, 47 years old, APACHE IV score = 41, 2 days on ICU	*Labetalol* ICU dosage: 3dd200 mg	ICU discharge: wrong dose	Patient was admitted to ICU after suffering a subarachnoidal bleeding. Labetalol 3dd200 mg was started (high blood pressure). After ICU discharge this dosage was by mistake reduced to 1dd50 mg. Blood pressure went up to > 180 mmHg	ICU discharge: 0.6
Female, 61 years old, APACHE IV score = 101, died after 15 days on ICU	*Valaciclovir* Dosage at home: 500 mg twice a day	ICU admission: omission	Valaciclovir used at home (prophylaxis after allogeneic bone marrow transplantation) was by mistake not prescribed at the hospital the patient was admitted to, prior to this ICU admission. This transfer error continued at ICU admission. During ICU stay the patient suffered graft versus host disease, aspergillosis and a herpes simplex infection and died due to multiorgan failure	ICU admission: 0.6
*Medium harm expected (nesbit = 0.4)*				
Female, 58 years old, APACHE IV = 97, 7 days on ICU	*Clozapine* Dosage at home: 8:00 am: 125 mg, 10:00 pm: 225 mg	ICU admission: omission	Clozapine (used at home) not prescribed to patient during ICU stay, no TDM monitoring performed	ICU admission: 0.4
		ICU discharge: different dose	Clozapine was restarted at a dose of 1dd350 mg, without TDM monitoring	ICU admission: 0.1
Male, 55 years old, APACHE IV score = 95, 7 days on ICU	*Nortriptyline* Dosage at home: 65 mg a.n.	ICU admission: different dose	Different doses of nortriptyline (depression) prescribed to patient during all transfers. Patient used 65 mg at home at home, 100 mg during ICU stay and 25 mg during stay at general ward	ICU admission: 0.4
		ICU discharge: different dose		ICU discharge: 0.4
	*Haloperidol* Dosage at home: 2 mg a.n.	ICU admission: omission	Haloperidol used at home was omitted. This omission started at admission and continued after ICU discharge	ICU admission: 0.4
		ICU discharge: omission		ICU discharge: 0.4
Male, 71 years old, APACHE IV score = 106, 39 days on ICU	*Colchicine* Dosage at home: 0.5 mg, once daily	ICU admission: omission	Colchicine not continued after ICU admission. During ICU stay the patient suffered a severe gout attack. After the gout attack colchicine 2 d.d. 0.5 mg was prescribed	ICU admission: 0.4
	*Allopurinol* Dosage at home: 100 mg, once daily	ICU admission: omission	Allopurinol not continued after ICU admission and discharge. See above	ICU admission: see above
		ICU discharge: omission		ICU discharge: 0.4
*Low harm expected (nesbit = 0.1)*				
Male, 74 years old, APACHE IV = 49, 6 days on ICU	*Dutasteride* Dosage at home: 0.5 mg, once daily	ICU discharge: omission	Dutasteride was omitted at ICU admission, restart after ICU discharge was forgotten	ICU discharge: 0.1
Female, 65 years old, APACHE IV score = 75, 4 days at ICU	*Esomeprazole* Dosage at ICU: 40 mg, once daily	ICU discharge: drug added	Esomeprazole was started at ICU and continued after ICU stay; however, there was no indication	ICU discharge: 0.1
*Very low harm expected (nesbit = 0.01)*				
Male, 55 years old, APACHE IV = 46, 3 days on ICU	*Salmeterol/flucticasone aerosol* Dosage at home: twice daily	ICU discharge: omission	Patient was treated for COPD and emphysema at home with salmeterol/fluticasone and tiotropium. At the ICU the patient was treated with ipratropium and salbutamol, 8 times a day. The first days after ICU discharge no COPD medication was given. At hospital discharge home medication was restarted	ICU discharge: 0.01
Male, 52 years old, APACHE IV = 128, 6 days on ICU	*SDD mouthpaste* (colistine, tobramycin AND amfotericin) ICU medication	ICU discharge: continued	Patient was treated with SDD mouthpaste (typical ICU medication) at the general ward for 1 day	ICU discharge: 0.01
*No harm expected (nesbit = 0)*				
Male, 59 years old, APACHE IV = 127, 6 days on ICU	*Calcium carbonate/colecalciferol* Dosage at home: 1000 mg/800 IE, a.n.	ICU admission: wrong dose	Patient got a 1000 mg/400 IE dosage at ICU and after ICU stay	ICU admission: 0
		ICU discharge: wrong dose		ICU discharge: 0
Female,71 years old, APACHE IV = 127, 5 days on ICU	*Ranitidine* ICU dosage: 150 mg, twice a day	ICU admission: drug added	Patient got ranitidine at ICU admission, since it was thought to be in use at home	ICU admission: 0

The demonstrated MTE is grouped based on their potential for harm (0.6 = high harm expected, 0.4 = medium harm expected, 0.1 = low harm expected, 0.01 = very low harm expected, 0 = no harm expected). All demonstrated MTEs were identified in the pre-intervention phase

*a.n.* ante noctem, *dd* daily dose, *ICU* intensive care unit, *SDD* selective decontamination of digestive tract, *TDM* therapeutic drug monitoring

Ref. [23]

## Abbreviations

APACHE IV: Acute Physiology and Chronic Health Evaluation II
ATC: Anatomical Therapeutic Chemical (a medication classification system)
BPMDL-ICU: best possible ICU medication discharge list
BPMH: best possible medication history
BPML-GW24: best possible general ward medication list 24 h after ICU discharge
CPOE/CDS: computerized physician order entry systems with clinical decision support
CRF: case report form
ER: emergency room
ER EPF: electronic patient file from the emergency room
HIS: hospital information system
ICU: intensive care unit
METC: medical ethics committee
MTE: medication transfer error
OR: odds ratio
pADE: *probable* adverse drug event
PDMS: patient data monitoring system
SAPS II: Simplified Acute Physiology Score
TIM: Transfer ICU and Medication reconciliation program
